# Meteorological and land-use determinants of *Culex pipiens s.l.* spatio-temporal dynamics in Northern Italy

**DOI:** 10.1093/jme/tjag094

**Published:** 2026-06-30

**Authors:** Anna Simonetto, Francesco Defilippo, Mattia Calzolari, Ana Maria Moreno Martin, Michele Dottori, Davide Lelli, Davide Monici, Andrea Pintossi, Antonio Lavazza, Gianni Gilioli

**Affiliations:** Department of Civil, Environmental, Architectural Engineering and Mathematics, Università degli Studi di Brescia, Brescia, Italy; Istituto Zooprofilattico Sperimentale della Lombardia e dell’Emilia Romagna, Brescia, Italy; Istituto Zooprofilattico Sperimentale della Lombardia e dell’Emilia Romagna, Reggio Emilia, Italy; Istituto Zooprofilattico Sperimentale della Lombardia e dell’Emilia Romagna, Brescia, Italy; Istituto Zooprofilattico Sperimentale della Lombardia e dell’Emilia Romagna, Reggio Emilia, Italy; Istituto Zooprofilattico Sperimentale della Lombardia e dell’Emilia Romagna, Brescia, Italy; Department of Civil, Environmental, Architectural Engineering and Mathematics, Università degli Studi di Brescia, Brescia, Italy; Department of Civil, Environmental, Architectural Engineering and Mathematics, Università degli Studi di Brescia, Brescia, Italy; Istituto Zooprofilattico Sperimentale della Lombardia e dell’Emilia Romagna, Brescia, Italy; Department of Civil, Environmental, Architectural Engineering and Mathematics, Università degli Studi di Brescia, Brescia, Italy

**Keywords:** *Culex pipiens s.l*, West Nile virus, environmental drivers, risk assessment, vector surveillance

## Abstract

Mosquito population dynamics are a key determinant of West Nile virus (WNV) transmission risk in temperate regions, as they regulate the timing, intensity, and spatial distribution of seasonal outbreaks. In Europe, *Culex pipiens s.l.* is the primary WNV vector, yet a comprehensive understanding of how its populations respond to environmental variability across multiple temporal and spatial scales remains limited. Using a nine-year entomological dataset (2014–2022) from an extensive monitoring network in Northern Italy, we investigated the spatio-temporal dynamics of *Cx. pipiens s.l.* across the heterogeneous landscapes of the Po Valley, a WNV-endemic area. We applied complementary statistical and machine-learning approaches to characterize between-year trends, within-year (summer) fluctuations, and long-term spatial patterns in mosquito abundance. Results showed strong persistence in population states across both years and months, consistently modulated by hydroclimatic conditions. Precipitation during the pre-activity period emerged as a dominant driver of inter-annual variability, highlighting its potential as an early indicator of summer population build-up. Within seasons, short-term temperature exerted a strong, nonlinear influence on *Cx. pipiens s.l.* abundance, with declines observed under extreme heat conditions. Spatial analyses identified persistent hotspots associated with irrigated agricultural systems, wetlands, and major river corridors, whereas upland and forest-dominated areas exhibited lower suitability. Overall, this study advances current knowledge of *Cx. pipiens s.l.* spatio-temporal dynamics and demonstrates how climatically and environmentally driven indicators can be translated into actionable tools for risk assessment and adaptive vector surveillance. These insights support improved early detection and targeted control within integrated One Health surveillance frameworks for WNV.

## Graphical Abstract

**Graphical Abstract:**
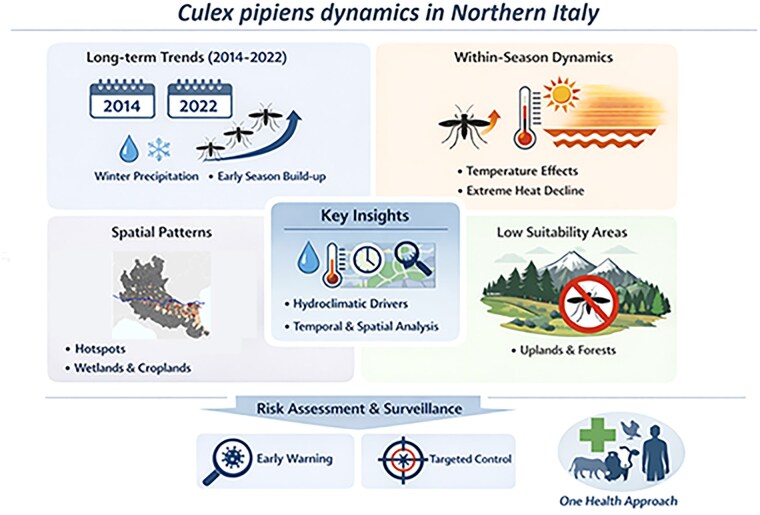

## Introduction

The common house mosquito, *Culex pipiens s.l.* Linnaeus, 1758, is a cosmopolitan species widely distributed across Europe, North America, North Africa, and parts of Asia. Recent evidence indicates a progressive northward expansion of its range, likely driven by climate warming (Balenghien et al. 2008). *Cx. pipiens s.l.* shows marked ecological plasticity and is able to exploit a broad variety of aquatic habitats, including artificial containers in urban settings as well as natural or semi-natural wetlands. The larval stages develop efficiently in stagnant water, while adults display predominantly ornithophilic feeding behavior, which supports virus amplification within avian hosts. Nevertheless, the species may also feed on mammals, including humans (Diaz-Badillo et al. 2011, Krida et al. 2015).

*Cx. pipiens s.l.* is the primary vector of West Nile virus (WNV) in temperate regions of the Northern Hemisphere. WNV is a widely distributed flavivirus belonging to the *Japanese encephalitis* serocomplex and is maintained in nature through an enzootic cycle involving *Culex* mosquitoes (primarily *Cx. pipiens s.l.*) and avian hosts, while humans and other mammals act as incidental, dead-end hosts (Giesen et al. 2023, Tolsá-García et al. 2023). WNV transmission is strongly seasonal worldwide; in Europe, transmission usually occurs from June to October with a peak in mid-summer (Young et al. 2021). The timing and intensity of seasonal circulation are shaped by environmental and climatic factors, vegetation ­patterns, and the availability of suitable aquatic habitats, which influence the distribution and activity of both vectors and hosts (Giesen et al. 2023). Extreme weather events, such as heatwaves and drought, can further modulate these dynamics by advancing or amplifying transmission (Pacenti et al. 2020).

Over recent decades, WNV activity has increased across Europe, with expansion into new geographic areas and recurrent outbreaks in both humans and animals. The 2018 transmission season was the most severe on record in Central and Southern Europe, with more than 2,000 reported symptomatic human cases (Barzon et al. 2022). Italy is among the most affected countries and is now considered endemic (Fornasiero et al. 2020, Mencattelli et al. 2022, Giesen et al. 2023). Endemic transmission is consistently reported in Emilia-Romagna, Lombardy, Veneto, and Sardinia, with recurrent seasonal outbreaks concentrated in the Po Valley, where ecological and climatic conditions are highly favorable (Mulatti et al. 2014, Mencattelli et al. 2022). Notably, both 2018 and 2022 were characterized by intense WNV transmission in Italy, with 606 reported human cases in 2018 and an unusually early onset and increased number of neuroinvasive cases in 2022, resulting in the highest WNV-related case and fatality counts recorded in the EU/EEA (Pacenti et al. 2020, Barzon et al. 2022). To address this persistent risk, Italy has established an integrated One Health surveillance system combining human, veterinary, and entomological monitoring, which enables early detection of seasonal WNV and Usutu virus circulation and supports timely public health responses (Riccardo et al. 2022). Climatic drivers, particularly temperature and precipitation, strongly influence *Cx. pipiens s.l.* population dynamics and WNV transmission. For example, the 2022 drought in the Po Valley likely concentrated vectors and hosts around limited water sources, facilitating virus amplification once favorable conditions returned (Pacenti et al. 2020, Riccardo et al. 2022).

Given the ecological plasticity of *Cx. pipiens s.l.* and its role as the primary vector of WNV in Europe, understanding the environmental and climatic factors that influence its population dynamics is essential for anticipating transmission risk and informing vector control strategies. Several studies have investigated the ecological determinants of *Cx. pipiens s.l.* distribution and abundance, often using climatic and land-use variables to model habitat suitability or population dynamics. In Italy, most research has focused on defined geographic areas and specific temporal windows, reflecting the structure of available surveillance data.

Long-term analyses in northwestern Italy have provided preliminary evidence that temperature, precipitation, and agricultural landscapes, particularly irrigated systems such as rice fields, shape seasonal dynamics of *Cx. pipiens s.l.* (Mughini-Gras et al. 2014, Marini et al. 2016). Similarly, weather-driven models in the Po Valley have suggested that thermal conditions and rainfall patterns contribute to interannual fluctuations in mosquito abundance (Carrieri et al. 2014). More recently, remote sensing and machine-learning approaches have improved the spatial resolution of habitat-suitability assessments; however, these methods have generally been applied to restricted areas or relatively short monitoring periods (Ippoliti et al. 2024, Liu et al. 2024). Despite these contributions, a comprehensive long-term assessment integrating spatial heterogeneity with both between- and within-year temporal dynamics is still lacking. Moreover, few studies jointly apply multiple statistical and machine-learning approaches alongside interpretability tools, limiting the ability to move beyond pattern detection toward a mechanistic understanding of the environmental drivers underlying *Cx. pipiens s.l.* population dynamics.

The present study addresses these gaps by leveraging a long-term dataset (2014–2022) derived from a dense monitoring network located in highly populated and ecologically important areas of Northern Italy, where favorable environmental conditions and intense human activity support both the persistence of *Cx. pipiens s.l.* and the rapid spatial spread of WNV. This dataset enables a joint assessment of between-year, within-year, and spatial variation in mosquito abundance across heterogeneous landscapes. Considering both interannual and intraseasonal dynamics is essential, as these reflect distinct ecological processes operating at different temporal scales: pre-season climatic conditions may influence early population build-up and processes occurring prior to the onset of the transmission season, while short-term weather variability regulates mosquito development, survival, and activity during the transmission season.

By explicitly integrating these temporal dimensions, the study provides a more comprehensive and mechanistic understanding of *Cx. pipiens s.l.* population dynamics compared to approaches focusing on a single temporal scale. By combining gradient boosting and random forest models with partial dependence plots and transition probability matrices, we generate outputs that link predictive performance with interpretability. This framework allows us not only to identify key environmental drivers across scales but also to translate them into actionable indicators for early warning and adaptive vector surveillance. Overall, this multiscale and multimethod approach advances current understanding of vector ecology and supports improved assessment of WNV transmission risk in endemic regions.

## Materials and Methods

### Sampling Design and Mosquito Collection

The surveyed area comprised the plain areas of the Lombardy and Emilia-Romagna regions, covering approximately 24,000 km^2^, corresponding to about half of the Po Valley and slightly more than 50% of the combined surface area of the two regions. This densely populated area (14.5 million people) includes numerous urban centers and represents one of the most anthropogenically modified landscapes in Italy. Rural areas are dominated by intensive agricultural and livestock practices, with limited hedgerows, sparse tree cover, and an extensive irrigation network. The climate is classified as continental, although local conditions vary over short distances due to topography, exposure to prevailing winds, and the proximity of large lake basins, which locally confer Mediterranean influences. Temperature regimes are strongly influenced by altitude and exposure. Summers are typically hot, with temperatures frequently exceeding 30 °C and often surpassing 35 °C during heatwaves in inland basins and across the Po Valley.

Entomological data analyzed in this study derive from routine mosquito surveillance conducted between 2014 and 2022 within the framework of the Italian national surveillance programme, in accordance with national guidelines for arbovirus surveillance (Ministero della Salute 2019). Surveillance was focused on rural areas and farms in Lombardy and on rural, seminatural, and peri-urban areas in Emilia-Romagna, ensuring coverage of the plain areas of both regions (Calzolari et al. 2020). All traps were located in plain areas or at elevations below 300 m a.s.l. Mosquito sampling was carried out fortnightly from June to October using modified CDC traps baited with carbon dioxide (CO_2_–CDC traps). Traps were supplied with dry ice pellets as a CO_2_ source to attract hematophagous insects and powered by a 12 V battery (Bellini et al. 2002).

**Fig. 1.**
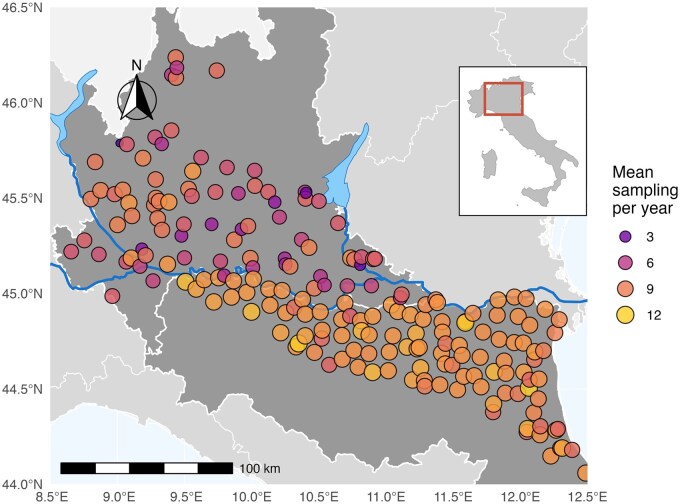
Map of the spatial distribution and mean sampling frequency per year of monitoring traps in Lombardy and Emilia-Romagna.

A total of 139 trapping sites were included in the study. In Lombardy, the number of traps increased progressively from 38 in 2014 to 44 by 2020 and was regularly distributed across the region using a 20 × 20 km grid. In Emilia-Romagna, the number of traps increased from 88 to 95 and were distributed according to an approximately 11 × 11 km grid (Fig. 1). All insects collected from each trap was retrieved and shipped to the IZSLER laboratories, where mosquitoes were identified morphologically using standard dichotomous keys (Becker et al. 2003, Severini et al. 2009).

### Meteorological Data

Temperature and precipitation were considered the main abiotic drivers influencing *Cx. pipiens s.l.* development, survival, and adult activity. To assess their effects across multiple temporal scales, we derived meteorological indicators (Table 1) capturing short-term variation, cumulative thermal and rainfall inputs, and early-season conditions influencing population build-up in temperate regions.

**Table 1.** Description and summary statistics of the meteorological variables used in the study^a^

Variable	Description	Unit	Mean (±SD)	Range	Between-year	Within-year	Spatial
**T**_24H_	Mean temperature in the 24 h prior to sampling	°C	22.70 ± 4.18	[8.63–33.36]		x	
**T**_14d_	Mean temperature in the 14 days prior to sampling	°C	22.77 ± 3.49	[11.96–30.16]		x	
**T**_24H_	Cumulative rainfall in the 24 h prior to sampling	mm	2.44 ± 6.57	[0–127.54]		x	
**R**_14d_	Cumulative rainfall in the 14 days prior to sampling	mm	36.89 ± 30.48	[0.05–261.76]		x	
**R**_24H,ov_	Number of hours with rainfall >1 mm/h in the 24 h prior to sampling	hours	0.67 ± 1.88	[0–18.00]		x	
**DD**_14,in_	Cumulative degree-days between 10 and 38 °C in the 14 days prior to sampling	°C	178.71 ± 48.42	[37.79–277.21]		x	
**DD**_14,ov_	Cumulative degree-days above 38 °C in the 14 days prior to sampling	°C	0.18 ± 1.49	[0–38.08]		x	
**DD**_PAP_	Cumulative degree-days between 10 and 38 °C in February, March, and April	°C	186.35 ± 32.55	[68.27–268.59]	x	x	x
**R**_PAP_	Cumulative rainfall in February, March, and April	mm	204.79 ± 90.13	[73.33–460.84]	x	x	x
**R**_Mayl_	Mean temperature in May	°C	17.71 ± 1.61	[13.31–20.29]			x
**T**_Jun_	Mean temperature in June	°C	23.31 ± 1.59	[19.20–26.76]			x
**T**_Jul_	Mean temperature in July	°C	20.68 ± 1.68	[25.75–28.91]			x
**R**_May_	Cumulative rainfall in May	mm	98.18 ± 50.27	[27.58–283.77]			x
**R**_Jun_	Cumulative rainfall in June	mm	70.03 ± 42.16	[69.41–283.64]			x
**R**_Jul_	Cumulative rainfall in July	mm	83.01 ± 63.41	[62.23–360.36]			x

a The final columns indicate the components of the spatio-temporal analyses (between-year, within-year, and spatial) to which each variable contributed.

Temperature was characterized using short-window averages ($T_{24H}$, $T_{14d}$), monthly averages ($T_{\mathrm{May}}$, $T_{\mathrm{Jun}}$, $T_{\mathrm{Jul}}$), and degree-day metrics. Among these, ${DD}_{\mathrm{PAP}}$ represents the cumulative degree-days (DD) accrued during the pre-activity period (PAP, defined as February–April) and is intended to capture thermal conditions that may influence early-season population dynamics, including processes related to overwintering and the onset of larval development and the timing and magnitude of the first seasonal population increase. ${DD}_{14,\mathrm{in}}$ represents cumulative degree-days accumulated between 10 °C and 38 °C during the 14 days preceding sampling, reflecting short-term thermal conditions favorable for mosquito development. ${DD}_{14,ov}$ represents cumulative degree-days accumulated at temperatures above 38 °C over the same period and was included to capture exposure to potentially stressful or overheating conditions. The 14-day window was selected to account for the influence of meteorological conditions on mosquito development and population dynamics prior to sampling, while the 24-h variables were included to capture immediate effects on adult behavior and trap efficiency, in line with previous studies conducted in similar ecological contexts (Gilioli et al. 2024).

Precipitation was described through short-term cumulative rainfall ($R_{24H}$_,_  $R_{14d}$), monthly ($R_{\mathrm{May}}$, $R_{\mathrm{Jun}}$, $R_{\mathrm{Jul}}$), and pre-activity period ($R_{\mathrm{PAP}}$) precipitation. $R_{\mathrm{PAP}}$ reflects rainfall conditions during the pre-activity period and may influence larval habitat availability prior to the onset of peak transmission. $R_{24H,ov}$ represents the number of hours with rainfall exceeding 1 mm h^−1^ during the 24 h preceding sampling and was included to capture the potential effects of intense precipitation events on adult mosquito activity and trap efficiency.

All meteorological variables were obtained from the ERA5-Land reanalysis dataset (Muñoz-Sabater et al. 2021), which provides hourly estimates at a spatial resolution of 0.1° × 0.1°. Site-specific values for each *Cx. pipiens s.l.* trapping site were calculated by applying an altimetric correction to the extracted gridded data, utilizing the high-resolution TINITALY Digital Elevation Model (Tarquini 2023).

### Land-Use Characterization

Land-use data for the study area were obtained from regional geodatabases. To account for landscape changes over the 9-year study period and avoid temporal mismatches, land-use maps were matched to the closest available year for each trapping period: 2015, 2018, and 2021 for Lombardy (Regione Lombardia, 2015–2021) and 2014, 2017, and 2020 for Emilia-Romagna (Regione Emilia-Romagna 2014–2020). To ensure complete spatial coverage for buffers near regional borders, land-use data from the adjacent Piedmont (Regione Piemonte, 2021) and Veneto (Regione Veneto, 2015–2021) were integrated. Although these datasets are largely based on the CORINE Land Cover classification, regional differences exist at finer levels. To ensure consistency, all datasets were harmonized into a unified land-use scheme. Following Gilioli et al. (2024), re functionally or structurally equivalent classes were matched, and selected Level 3 categories were aggregated where appropriate. The resulting harmonized classification includes 17 land-use categories representing both natural and anthropogenic habitats (Table 2).

**Table 2.** Land-use categories and their proportional coverage within a 2,000 m buffer surrounding each sampling site^a^

Land-use category	Mean (±SD)	Range [min–max]	Land-use category	Mean (±SD)	Range [min–max]
Agricultural production units	1.14 ± 0.87	[0.00–4.58]	Pastures	2.54 ± 5.20	[0.00–31.81]
Annual crops	58.43 ± 21.52	[0.47–93.93]	Permanent crops	6.81 ± 8.62	[0.00–52.28]
Forests	2.63 ± 6.21	[0.00–52.93]	Rice fields	2.05 ± 9.29	[0.00–74.34]
Graveyard	0.10 ± 0.17	[0.00–1.57]	Scarce vegetation	0.15 ± 0.62	[0.00–7.07]
Green urban areas	1.53 ± 3.20	[0.00–35.90]	Sport and leisure	0.73 ± 1.29	[0.00–11.56]
Industrial/commercial units	4.28 ± 5.99	[0.00–40.59]	Transitional woodland/shrub	1.16 ± 1.35	[0.00–8.10]
Infrastructures	2.44 ± 3.47	[0.00–38.18]	Urban areas	8.25 ± 6.85	[0.11–37.02]
Marshes	1.94 ± 7.05	[0.00–58.99]	Water bodies	4.91 ± 5.35	[0.00–24.76]
Mine and construction sites	0.88 ± 1.59	[0.00–15.19]			

a Values are expressed as percentages of buffer area and summarized across all sampling sites.

To characterize the landscape surrounding each *Cx. pipiens s.l.* sampling site, a circular buffer with a radius of 2,000 m was employed. This spatial extent was specifically selected to reflect the biologically relevant dispersal capacity of the vector. Mark–recapture studies report mean dispersal distances of approximately 1.3 km and maximum distances up to 1.98 km (Ciota et al. 2012), while flight capacity studies indicate that adult females can exceed 2 km under controlled conditions (Cui et al. 2013). Taken together, these findings support the use of a 2 km buffer as an ecologically relevant spatial scale.

### Temporal Population Dynamics

Temporal dynamics of *Cx. pipiens s.l.* abundance were analyzed using a two-step approach: between-year analyses focused on long-term population trends, whereas within-year models examined short-term fluctuations during the main summer activity period (June–August). To model these dynamics, tree-based ensemble methods, specifically gradient boosting and random forest, were selected over traditional parametric approaches. These techniques were chosen for their superior ability to automatically capture complex, nonlinear ecological relationships and high-order interactions among predictors without requiring prior specification (Prasad et al. 2006,  Elith et al. 2008).

Two complementary modeling techniques were applied at each step. First, a gradient boosting classifier (Friedman 2001) was employed due to its high predictive accuracy to derive transition probability matrices. This approach captured probabilistic changes in abundance across years or months conditioned on specific climatic scenarios (temperature and precipitation). Second, a random forest model (Breiman 2001) was chosen for its inherent robustness against overfitting to quantify the relative importance of a broader set of environmental predictors, including both climatic and land-use variables. Finally, Partial Dependence Plots (PDPs) were generated to interpret the effects of individual predictors within the Random Forest framework, visualizing their marginal influence while accounting for the average effects of all other variables. Together, these analyses provide an integrated assessment of the environmental drivers shaping *Cx. pipiens s.l.* abundance across temporal scales. To ensure robust estimates and consistent sampling effort, the dataset was restricted to site–year combinations with a minimum of six sampling events across the three summer months.

#### Between-Year Dynamics

Between-year dynamics were analyzed to assess how pre-activity period climatic conditions and landscape structure influence year-to-year changes in *Cx. pipiens s.l.* abundance, reflecting early-season processes, including those related to overwintering conditions and the onset of spring population growth. Summer mean abundances were grouped into three classes (A_1_, A_2_, and A_3_) based on tertiles to define discrete population states for transition probability analysis. Pre-activity period precipitation (*R*_PAP_) and cumulative degree-days (DD_PAP_), two abiotic factors known to affect diapause termination, early larval habitat availability, and subsequent adult emergence, were likewise categorized into tertiles (RW_1_, RW_2_, RW_3_, DD_1_, DD_2_, and DD_3_) to assess their combined effects on annual population trajectories. Transition matrices were estimated using a gradient boosting classifier, providing a probabilistic approach to examine how pre-activity period climatic scenarios shape between-year dynamics.

A Random Forest model was used to predict mosquito abundance as a function of continuous pre-activity period climatic predictors and harmonized land-use variables to assess the role of climatic conditions and landscape composition in shaping between-year patterns. For this analysis, mosquito abundance was expressed as a ranked index to emphasize spatial patterns while reducing sensitivity to year-specific absolute values.

#### Within-Year Dynamics

Within-year dynamics were analyzed to assess how short-term climatic conditions and landscape composition influence intra-seasonal changes in *Cx. pipiens s.l.* abundance within a single transmission season. In contrast to the between-year analysis, the response variable consisted of abundance observations at each sampling date, allowing monthly fluctuations during the peak activity period (June–August) to be examined.

Monthly mean temperature ($T_{\mathrm{Jun}}$, $T_{\mathrm{Jul}}$) and precipitation ($R_{\mathrm{Jun}}$, $R_{\mathrm{Jul}}$) were categorized into tertiles ($T_{Jn,1}-T_{Jn,3}$, $T_{Jl,1}-T_{Jl,3}$, $R_{Jn,1}-R_{Jn,3}$, $R_{Jl,1}-R_{Jl,3}$, respectively) to define discrete environmental states for transition probability analysis. Transitions from June to July and from July to August were modeled using a Gradient Boosting classifier, providing a probabilistic framework to evaluate how contrasting summer climatic conditions shape within-year dynamics.

A Random Forest model was used to predict monthly *Cx. pipiens s.l.* abundance as a function of continuous summer climatic predictors, pre-activity period climatic variables representing early-season conditions, and harmonized land-use variables. The response variable was defined as the mean number of mosquitoes collected per trap in each month (June, July, or August).

### Spatial Abundance

Spatial patterns of *Cx. pipiens s.l.* abundance were analyzed to assess the environmental drivers (meteorological conditions and land-use composition) underlying long-term spatial variability at the trap level. The response variable was defined for each sampling site as the mean number of mosquitoes collected per trap during the peak activity period (June–August), averaged over the nine-year monitoring period (2014–2022). This site-specific metric was used as an indicator of long-term ­mosquito abundance.

To address potential multicollinearity among environmental predictors and ensure model parsimony, an Elastic Net regularization approach was used for variable selection (Zou and Hastie 2005). An Ordinary Least Squares (OLS) regression model was then fitted using the selected variables. Spatial autocorrelation in model residuals was assessed using Global Moran’s *I* with a k-nearest neighbors spatial weights matrix (Bivand and Wong 2018). As significant spatial dependence was detected, a Spatial Error Model (SEM) was applied to obtain unbiased coefficient estimates and 95% confidence intervals (Anselin 1988). A second Moran’s *I* test on SEM residuals confirmed the removal of spatial autocorrelation.

To visualize spatial patterns at an administrative scale relevant for public health applications, a municipal-level abundance map was subsequently generated by averaging the site-specific risk values across all traps located within each municipality.

### Statistical Analysis

All statistical analyses were performed in R (version 4.4.1). Prior to models fitting, the response variable was $\log_{10}$-transformed to reduce skewness and improve residual normality. Predictor variables were standardized to facilitate model convergence and enable comparison of effect effects.

Machine-learning models were trained and tuned using the caret framework (Kuhn 2008). Gradient Boosting models were implemented using the gbm package (Ridgeway and Developers 2003). As the approach was strictly predictive, multicollinearity among predictors was not constrained. Hyperparameters (interaction.depth, n.minobsinnode, shrinkage, n.trees) were optimized via grid search. Model performance was evaluated using standard 5-fold cross-validation, with logarithmic loss (logLoss) as the optimization metric.

Prior to the Random Forest analysis, multicollinearity among predictors was assessed using Spearman’s rank correlations and Variance Inflation Factors (VIF), with highly collinear variables excluded. Random Forest regression models were implemented using the ranger package (Wright and Ziegler 2017). Model performance was evaluated using 10-fold spatial cross-validation, grouping observations by trapping site to prevent spatial data leakage. Hyperparameters (mtry and min.node.size) were optimized via grid search, with Root Mean Squared Error (RMSE) serving as the optimization metric.

For the spatial abundance analysis, Elastic Net regularization was implemented using the glmnet package (Friedman et al. 2010), with model tuning performed through 10-fold cross-validation. Global Moran’s *I* and the Spatial Error Model (SEM) were implemented using the *spdep* and *spatialreg* packages (Bivand et al. 2013), based on a 10-nearest neighbors spatial weights matrix.

## Results

The final dataset comprises *Cx. pipiens s.l.* surveillance records collected between 2014 and 2022 across two northern Italian regions, Lombardy and Emilia-Romagna. Each year, 118 to 139 traps were monitored, with Emilia-Romagna consistently contributing the larger share (88–95 traps annually). In Lombardy, the number of active traps increased gradually from 30 in 2014 to 44 in 2022 (Table 3 and Supplementary Appendix S1 and Table A1).

**Table 3.** Trapping effort and temporal coverage of *Cx. pipiens s.l.* surveillance in Lombardy and Emilia-Romagna (2014–2022)

	Lombardy				Emilia-Romagna					
Year	N° traps	Mean sampling events	First sampling date	Last sampling date	N° traps	Mean sampling events	First sampling date	Last sampling date	N° traps	Mean sampling events
2014	30	9	16/05	24/09	88	8.8	27/05	01/10	118	8.9
2015	33	7.9	09/06	01/10	88	8.5	04/06	07/10	121	8.2
2016	32	7.9	06/07	30/09	93	9.2	07/06	12/10	125	8.55
2017	36	8.8	31/05	07/11	94	6.7	13/06	09/10	130	7.75
2018	40	9.1	05/06	27/10	95	9	12/06	16/10	135	9.05
2019	41	7.5	30/05	31/10	95	10.9	14/05	11/10	136	9.2
2020	43	5	03/06	02/10	95	12	05/05	15/10	138	8.5
2021	44	6.4	03/06	04/10	95	12	04/05	14/10	139	9.2
2022	44	5.3	30/05	26/10	95	12	03/05	13/10	139	8.65

The number of sampling events per site per year showed moderate between-year variability. In Lombardy, the mean number of samplings per site ranged from 5.0 to 9.1, whereas Emilia-Romagna showed a broader range (6.7 to 12), reflecting the expansion of the regional monitoring programme from 2019 onwards. When combining both regions, the mean annual sampling frequency per site varied between 7.75 and 9.2 across the study period.

The temporal extent of surveillance followed a consistent seasonal pattern, with first captures using monitoring traps occurring between early May and early June, depending on operational schedules. However, Culex mosquitoes may be present earlier in the season but are not detected due to the absence of active surveillance. Last observations were generally recorded between late September and late October; only in 2017 did sampling extend into early November.

**Fig. 2.**
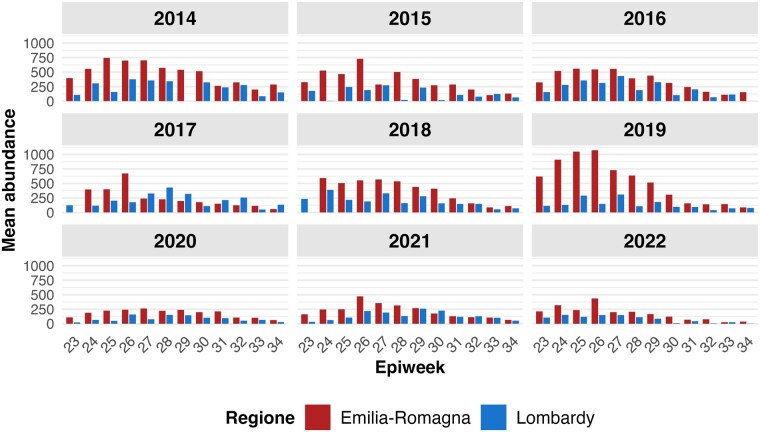
Weekly mean *Cx. pipiens s.l*. abundance during the summer months (Epiweeks 23–34) in Lombardy and Emilia-Romagna from 2014 to 2022.

Figure 2 illustrates the weekly mean *Cx. pipiens s.l.* abundance during the summer months (expressed by epidemiological week, from week 23 to 34) for Lombardy and Emilia-Romagna between 2014 and 2022. The intra-annual dynamics highlight a distinct seasonal curve, with captures typically peaking between epiweeks 25 and 28 before gradually declining toward the end of the summer. Throughout the study period, Emilia-Romagna consistently recorded higher mean weekly abundances compared to Lombardy. Significant between-year variability is also evident: peak summer abundances were generally high between 2014 and 2019, culminating in a pronounced maximum during the 2019 season in Emilia-Romagna. Following this peak, a marked overall decline in weekly capture rates is observable from 2020 onwards across both regions. This recent reduction may reflect a combination of environmental variability, climatic factors, and adjustments in regional surveillance protocols.

### Between-Year Dynamics

The transition matrices obtained from the gradient boosting model (Table 4) show that pre-activity period climatic conditions substantially influence the between-year dynamics of *Cx. pipiens s.l.* abundance. Each matrix cell represents the estimated probability of transitioning from one abundance class in a given year to another class in the subsequent year under specific climatic conditions. While a pattern of state persistence is visible (highlighted by the grey cells along the main diagonals), it is not uniform across all states. Strong persistence is primarily observed for the lowest abundance class (A_1_), particularly under dry pre-activity period conditions (RW_1_), where populations have an 80–86% probability of remaining low. Conversely, medium (A_2_) and high (A_3_) abundance classes exhibit significant volatility and climate-driven transitions. The interaction between pre-activity period temperature and precipitation clearly modulates the direction of year-to-year changes. Under drier pre-activity period conditions, there is a strong downward pressure on population size; for instance, a warmer but drier pre-activity period (RW_1_-DD_3_) forces high-abundance sites (A_3_) to transition to lower classes in 73% of cases. In contrast, wetter pre-activity periods (RW3) not only favor upward transitions (eg A_2_ to A_3_) but also strongly stabilize populations already at high abundance, with A_3_ to A_3_ persistence reaching up to 73% in RW_3_-DD_2_.

**Table 4.** Between-year transition matrices for *Cx. pipiens s.l.* abundance classes under different pre-activity period climatic scenarios^a^

		DD1			DD2			DD3		
		A1	A2	A3	A1	A2	A3	A1	A2	A3
RW1	A1	0.81	0.16	0.03	0.86	0.11	0.03	0.80	0.16	0.05
	A2	0.39	0.44	0.17	0.51	0.35	0.14	0.36	0.38	0.26
	A3	0.17	0.45	0.38	0.16	0.48	0.35	0.28	0.45	0.27
RW2	A1	0.65	0.25	0.10	0.75	0.17	0.08	0.59	0.23	0.18
	A2	0.19	0.51	0.30	0.29	0.42	0.29	0.13	0.33	0.54
	A3	0.06	0.33	0.61	0.08	0.30	0.62	0.54	0.34	0.12
RW3	A1	0.54	0.35	0.11	0.56	0.31	0.13	0.53	0.32	0.14
	A2	0.15	0.56	0.28	0.17	0.51	0.32	0.14	0.44	0.42
	A3	0.05	0.24	0.81	0.04	0.24	0.73	0.11	0.28	0.61

a Each cell represents the probability of transitioning from one abundance class to another between consecutive years. Grey shading indicates state persistence, while off-diagonal cells denote class transitions. Degree-days in the pre-activity period (DD), pre-activity period precipitation (RW), and summer mean abundance (A) are categorized into tertiles (1 = low, 2 = medium, 3 = high).

The model achieved an overall accuracy of 0.585 [Kappa = 0.3765, *P* < 0.001 vs NIR, confusion matrix Supplementary Appendix S1 and Table A2], supporting its capacity to capture climate-driven annual shifts in abundance.

**Fig. 3.**
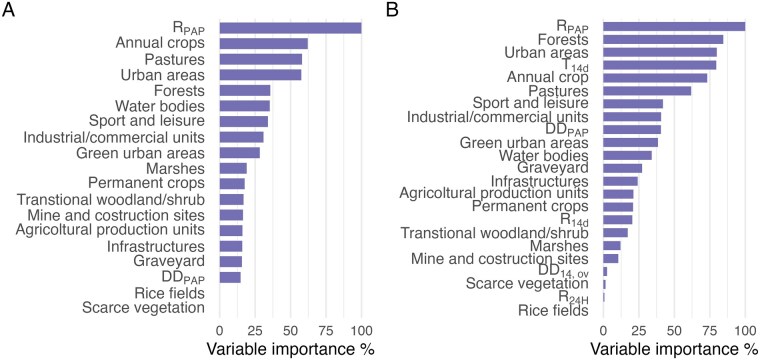
Variable importance from Random Forest models predicting *Cx. pipiens s.l*. abundance based on climatic conditions and site-level land-use characteristics. The left panel (A) refers to the between-year model predicting mean annual abundance per trap, while the right panel (B) refers to the within-year model predicting mean monthly abundance per trap during the summer season.

The Random Forest analysis (Fig. 3-left) identified the key environmental predictors associated with between-year variability in *Cx. pipiens s.l.* abundance. Prior to modeling, collinearity among environmental predictors was assessed; no variables were excluded from the analysis as all pairwise Spearman correlation coefficients were ≤0.71 and Variance Inflation Factors (VIF) were ≤3.58, both remaining below the conventional exclusion thresholds. The model explained 53.5% of the variance in the response variable, namely the between-year abundance index, indicating good predictive performance and highlighting the combined influence of pre-activity period climatic conditions and landscape structure on year-to-year population dynamics. The pre-activity period cumulative precipitation emerged as the most influential predictor (incMSE% = 100), indicating that moisture availability during late winter and early spring periods plays a dominant role in shaping annual abundance patterns. Land-use variables followed in importance, with annual crops, pastures, and urban areas showing the strongest associations with mosquito abundance. Intermediate importance values were observed for forested areas, water bodies, and sport and leisure areas, whereas industrial/commercial units, green urban areas, and wetlands contributed relatively little to model performance. Sparsely vegetated areas and rice fields showed the lowest importance, indicating a limited role in explaining between-year variability in this context. Notably, pre-activity period degree days also showed low predictive importance in this specific between-year model.

**Fig. 4.**
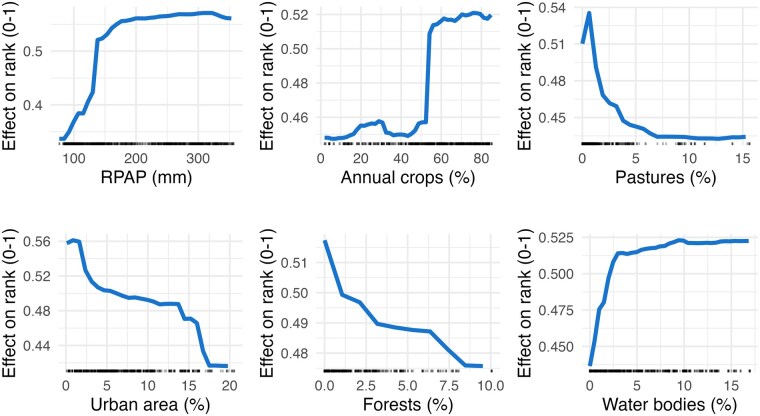
Partial dependence plots from the random forest between-year analysis predicting mean annual *Cx. pipiens s.l*. abundance per trap. Each plot shows the marginal effect of an environmental predictor on the predicted abundance while holding all other variables constant.

Partial dependence plots (Fig. 4) revealed the nonlinear functional forms of these relationships. Pre-activity period cumulative precipitation (R_PAP_) showed a strong positive association with the predicted abundance index, with values increasing sharply up to approximately 170 mm before reaching a stable plateau. Regarding land-use predictors, the effects varied significantly in shape and direction. Annual crops and water bodies exhibited clear positive associations. Specifically, annual crops displayed a distinct threshold effect: the predicted response remained relatively low and stable at lower percentages but exhibited a steep increase at approximately 50% coverage, remaining high thereafter. Water bodies demonstrated a rapid initial positive effect, increasing sharply and stabilizing at around 3–4% land coverage. Conversely, increasing proportions of pastures, urban areas, and forests were consistently associated with reduced predicted abundance. The negative effect of pastures was most pronounced at very low coverages, dropping steeply up to approximately 5% before leveling off at a minimum value. Urban areas showed a progressive, step-wise decline, culminating in a further sharp drop when coverage approached 15–17%. Finally, forested areas displayed a continuous and steady negative trend across the entire evaluated range (0–10% cover).

### Within-Year Dynamics

To characterize short-term fluctuations in *Cx. pipiens s.l.* abundance during the summer, month-to-month transitions between abundance classes were analyzed for the periods June–July and July–August (Table 5). While a degree of state persistence exists, particularly for the lowest abundance class (A_1_), the matrices reveal highly dynamic intra-seasonal shifts strongly driven by summer temperatures.

**Table 5.** Within-year transition matrices for *Cx. pipiens s.l.* abundance classes from June to July (left) and from July to August (right)^a^

Transition from June to July											Transition from July to August										
		$T_{Jn,1}$			$T_{Jn,2}$			$T_{Jn,3}$					$T_{Jl,1}$			$T_{Jl,2}$			$T_{Jl,3}$		
		A1	A2	A3	A1	A2	A3	A1	A2	A3			A1	A2	A3	A1	A2	A3	A1	A2	A3
**R**_Jn,1_	A1	0.62	0.27	0.11	0.69	0.25	0.06	0.75	0.22	0.03	**R**_Jl,1_	A1	0.39	0.44	0.17	0.59	0.34	0.07	0.70	0.26	0.04
	A2	0.18	0.43	0.39	0.25	0.48	0.27	0.32	0.50	0.18		A2	0.07	0.36	0.57	0.18	0.45	0.37	0.28	0.46	0.25
	A3	0.03	0.18	0.79	0.05	0.26	0.69	0.09	0.34	0.57		A3	0.01	0.17	0.82	0.05	0.27	0.68	0.09	0.35	0.56
**R**_Jn,2_	A1	0.68	0.23	0.09	0.74	0.21	0.05	0.79	0.18	0.03	**R**_Jl,2_	A1	0.51	0.39	0.10	0.69	0.27	0.04	0.78	0.20	0.02
	A2	0.22	0.40	0.38	0.29	0.45	0.26	0.37	0.46	0.17		A2	0.13	0.42	0.45	0.27	0.48	0.25	0.40	0.44	0.16
	A3	0.04	0.18	0.78	0.07	0.25	0.68	0.11	0.32	0.57		A3	0.03	0.23	0.74	0.09	0.35	0.56	0.15	0.41	0.44
**R**_Jn,3_	A1	0.59	0.30	0.11	0.66	0.28	0.06	0.72	0.25	0.03	**R**_Jl,3_	A1	0.62	0.29	0.09	0.78	0.19	0.03	0.86	0.13	0.01
	A2	0.17	0.46	0.37	0.23	0.52	0.25	0.29	0.54	0.17		A2	0.18	0.37	0.45	0.37	0.39	0.24	0.52	0.34	0.14
	A3	0.03	0.20	0.77	0.05	0.28	0.67	0.08	0.37	0.55		A3	0.04	0.20	0.76	0.13	0.30	0.57	0.22	0.35	0.43

a Each cell represents the probability of transitioning from one abundance class to another between consecutive months. Grey shading highlights state persistence along the diagonal, while off-diagonal cells indicate transitions to higher or lower abundance classes. Monthly mean temperature (TJn for June and TJl for July), monthly precipitation (RJn for June and RJl for July), and monthly mean abundance (A) are categorized into tertiles (1 = low, 2 = medium, 3 = high).

In both transition periods, high temperatures (T_3_) act as a severe limiting factor. Under these warmer conditions, the probability of populations persisting at high densities (A_3_ to A_3_) declines substantially, and downward transitions become much more frequent. For instance, during the July-August transition under hot conditions (T_3_), the probability of dropping from high to medium abundance (A_3_ to A_2_) ranges from 35% to over 40%. Conversely, milder thermal conditions (T_1_) systematically favor upward transitions (eg A_2_ to A_3_) and robustly stabilize populations already at peak abundance.

The interaction with precipitation is complex and varies between the two periods. During the early summer transition (June–July), temperature is the dominant driver, with precipitation playing a marginal role. However, later in the season (July–August), the combination of extreme heat and high precipitation (R_3_-T_3_) results in the highest probability of population collapse, with a 52% chance of transitioning from medium (A_2_) to low (A_1_) abundance, potentially reflecting the effect of intense summer storms on larval habitats. Overall, the data indicate that rather than stabilizing later in the summer, high-density populations become increasingly vulnerable to collapse under extreme late-summer heat.

The Gradient Boosting models achieved accuracies of 0.605 [Kappa = 0.4084, *P* < 0.001 vs NIR, confusion matrix Supplementary Appendix S1 and Table A3] for the June–July transition and 0.576 [Kappa = 0.3647, *P* < 0.001 vs NIR, confusion matrix Supplementary Appendix S1 and Table A4] for the July–August transition, indicating comparable predictive performance across summer months.

Prior to modeling the summer monthly dynamics, collinearity among predictors was assessed. The variable representing mean temperature in the 24 h before sampling (T_24H_) was excluded from the analysis due to a high Spearman correlation coefficient (0.77) with the mean temperature of the previous 14 days (T_14d_). For the remaining retained variables, the maximum Variance Inflation Factor (VIF) was 3.60, confirming the absence of severe multicollinearity. Random Forest analysis (Fig. 3-right) highlighted a strong effect of early-season moisture, with pre-activity period cumulative precipitation emerging again as the most critical predictor. Interestingly, landscape structure proved to be highly influential: forested areas, urban areas, annual crops, and pastures ranked among the top predictors. Recent thermal conditions also played a key role, with the mean temperature over the previous 14 days (T14D) acting as the most important short-term weather driver. Other environmental and climatic variables, including pre-activity period degree days, water bodies, and cumulative rainfall over the previous 14 days (R14D), contributed more moderately to the model performance. Conversely, strictly short-term weather events, such as precipitation in the 24 h prior to sampling (R24H), along with specific land covers like sparsely vegetated areas and rice fields, exhibited negligible importance in explaining month-to-month abundance variations during the summer.

**Fig. 5.**
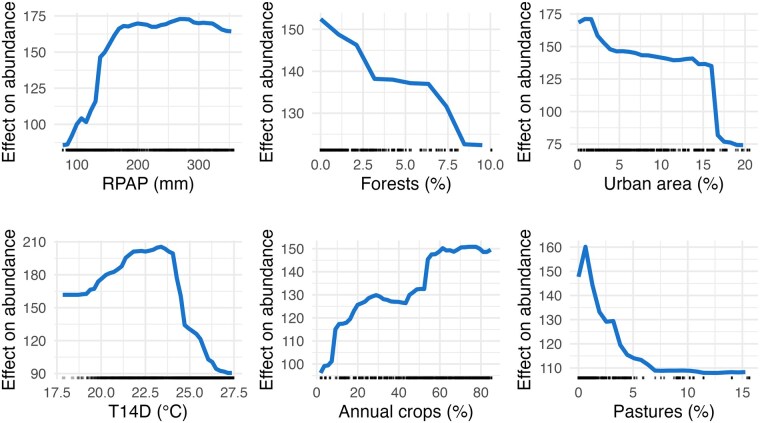
Partial dependence plots from the Random Forest within-year analysis predicting mean monthly *Cx. pipiens s.l*. abundance per trap for the summer months. Each plot shows the marginal effect of a predictor on the predicted abundance while holding all other variables constant.

The Partial Dependence Plots (PDPs) for the top-ranking predictors (Fig. 5) revealed distinct nonlinear relationships with predicted *Cx. pipiens s.l.* abundance. Pre-activity period cumulative precipitation (R_PAP_) showed a steep positive effect up to approximately 150–160 mm, after which the predicted abundance largely stabilized. Mean temperature in the 14 days prior to sampling (T_14d_) displayed a clear positive effect up to an optimal range of approximately 22.5–24 °C, followed by a sharp and continuous decline at higher temperatures.

Among the land-use variables, the proportion of annual crops demonstrated a predominantly positive association, with predicted abundance increasing as crop cover expanded, eventually plateauing at higher percentages (>60%). Conversely, increasing coverage of forests, urban areas, and pastures was associated with reduced predicted abundance, though with different functional forms. The negative effect of pastures was steepest at low cover percentages (dropping sharply up to ∼5%), whereas forest cover showed a more gradual, steady decline. Finally, urban areas maintained a moderate negative effect at low-to-intermediate coverages before exhibiting a sharp, precipitous drop when coverage exceeded approximately 15%.

### Spatial Abundance

The Elastic Net regularization (*α*  =  0.55, *λ*_1_se = 0.269) reduced the initial set of 25 environmental predictors to a parsimonious subset of 6 variables, achieving a cross-validated RMSE of 0.8317. An initial Ordinary Least Squares (OLS) regression fitted with these selected variables exhibited significant positive spatial autocorrelation in its residuals (Moran’s *I* = 0.1776, *P* < 0.001), indicating a violation of the independence assumption.

To account for this spatial dependence, a Spatial Error Model (SEM) was implemented. The SEM improved model fit, reducing the AIC from 433.45 (OLS) to 409.82 and yielded a McFadden pseudo-*R*^2^ of 0.2928. The spatial autoregressive coefficient was significant (*λ*  =  0.6457, *P* < 0.001). A subsequent Moran’s *I* test on the SEM residuals indicated that spatial autocorrelation was no longer present (Moran’s *I* = 0.0201, *P* = 0.1991).

Based on the spatial error model results (Table 6), four of the six retained environmental predictors exhibited effects on long-term *Cx. pipiens s.l.* abundance, with 95% confidence intervals not overlapping zero. Pre-activity period cumulative degree-days (DDPAP) showed a positive association with mosquito populations. Conversely, land-use categories including forests, green urban areas, and general urban areas were negatively associated with population levels. Because all continuous predictors were standardized prior to modeling, coefficient magnitudes reflect their relative influence. Accordingly, the most influential predictors were forests (*β* = −0.396) and green urban areas (*β* = −0.224).

**Table 6.** Spatial Error Model (SEM) analysis of spatial variation in *Culex pipiens s.l.* abundance using mean summer (June–August) mosquito counts per trap averaged across 2014–2022 as the response variable

Description	Beta	95% CI
Forest	−0.3957	[−0.5394; −0.2520]
Green urban area	−0.2237	[−0.3528; −0.0946]
DD_PAP_	0.1497	[0.0151; 0.2843]
Urban area	−0.1442	[−0.2746; −0.0139]
Water bodies	0.0932	[−0.0133; 0.1997]
Pastures	−0.0428	[−0.1703; 0.0847]

**Fig. 6.**
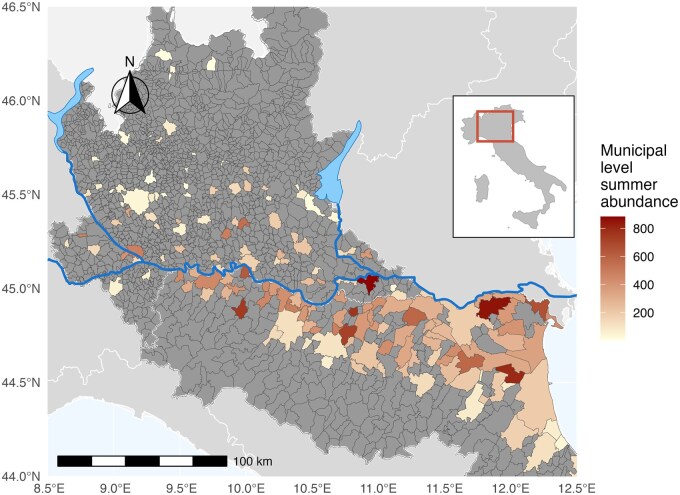
Spatial distribution of *Cx. pipiens s.l*. abundance across municipalities in Lombardy and Emilia-Romagna. For each municipality, abundance is expressed as a municipal-level abundance index defined as the mean *Cx. pipiens s.l*. abundance per trap per sampling date, averaged across all sampling sites within municipal boundaries and over the summer months (June–August) of the observation period (2014–2022). Municipalities shown in grey were not included in the surveillance programme and were therefore not considered in this study.

Model outputs were subsequently aggregated at the municipal level to generate a spatial representation of *Cx. pipiens s.l.* abundance (Fig. 6). For each municipality, a municipal-level abundance index was defined as the mean number of mosquitoes per trap per sampling date, averaged across all sampling sites within municipal boundaries. The resulting map shows clear geographic gradients, with higher abundance values concentrated along the Po River corridor, particularly in the central and eastern sectors of the basin, whereas upland and pre-alpine municipalities consistently exhibited lower levels.

## Discussion

### Between-Year Dynamic

Between-year analyses showed that *Cx. pipiens s.l.* population dynamics are strongly influenced by pre-activity period climatic conditions, confirming the key role of the pre-season period in shaping mosquito abundance in the subsequent summer. Transition matrix results indicated that warmer but drier pre-activity periods were associated with a higher probability of downward transitions in abundance, suggesting that low-moisture conditions may limit early-season habitat availability and constrain population build-up. These findings are consistent with previous studies reporting that winter aridity can constrain larval habitat persistence and hinder population recovery (Fornasiero et al. 2020, Bakran-Lebl et al. 2023).

In contrast, warmer and wetter pre-activity periods increased the likelihood of upward transitions, indicating that mild temperatures combined with sufficient precipitation favor early larval development and support higher mosquito abundance later in the season. This pattern is in line with earlier work emphasizing the importance of humid pre-season conditions for *Cx. pipiens s.l.* population growth and persistence (Mulatti et al. 2014, Rosà et al. 2014, Shutt et al. 2022).

Results from the random forest analysis further corroborated these patterns, identifying pre-activity period cumulative precipitation as the dominant driver of between-year variability in *Cx. pipiens s.l.* abundance. Land-use characteristics, particularly the presence of water bodies, pastures, and urban areas, also contributed, highlighting the combined influence of climatic conditions and landscape structure on annual population dynamics. Collectively, these findings indicate that *Cx. pipiens s.l.* is favored by humid environments characterized by moderate to high pre-activity period precipitation, limited forest cover, and the availability of aquatic and agricultural habitats, in agreement with the species’ established ecological preferences (Marini et al. 2016, Joyce et al. 2018, Amdouni et al. 2022). Although forest cover showed a negative association in this study, previous research has documented context-dependent effects of forested landscapes on *Cx. pipiens s.l.* abundance, likely driven by differences in habitat configuration, microclimatic conditions, and host availability (Giesen et al. 2023, Krol et al. 2023).

From an applied perspective, these between-year relationships have direct implications for early-season risk assessment. Pre-activity period anomalies in temperature and precipitation, particularly deviations in cumulative rainfall, may serve as early indicators of potential mosquito population build-up and increased arbovirus amplification risk. Integrating such climatic signals into forecasting frameworks could support public health authorities in anticipating high-risk seasons and optimizing surveillance intensity, trap allocation, and entomological testing prior to the onset of summer transmission.

### Within-Year Dynamics

Within-year analyses confirmed that climatic variability is the primary driver of *Cx. pipiens s.l.* abundance during the summer season, with both early-season moisture availability and short-term temperature conditions exerting strong effects. Pre-activity period precipitation and mean temperature in the 14 days preceding sampling emerged as the most influential predictors, indicating that moisture availability during late winter and early spring, together with recent thermal inputs, jointly modulates summer mosquito activity (Fornasiero et al. 2020, Moser et al. 2023). Random Forest results revealed pronounced nonlinear responses, with mosquito abundance declining sharply at mean temperatures above approximately 24 °C. This pattern is consistent with field evidence indicating a thermal optimum for *Cx. pipiens s.l.* around 23–24 °C, beyond which extreme heat negatively affects population persistence and survival (Lim et al. 2021). These findings are consistent with the established role of accumulated degree-days and near-term thermal fluctuations in shaping mosquito emergence, development, and biting behavior (Mulatti et al. 2014, Calzolari et al. 2020). Temperature during the two weeks preceding sampling affects both larval development and adult abundance (Fornasiero et al. 2020), while short-term thermal maxima influence viral infection rates (Calzolari et al. 2020). Mechanistic models similarly demonstrate the predictive value of daily temperature and photoperiod (Shutt et al. 2022).

The role of recent rainfall remains context-dependent: while some studies identified precipitation as a key driver of summer abundance (Bakran-Lebl et al. 2023), others reported weak or inconsistent effects in irrigated landscapes where water availability is largely decoupled from rainfall (Calzolari et al. 2020, Fornasiero et al. 2020). In such settings, hydrological indicators such as water-level measurements may provide more reliable proxies for larval habitat availability (Shutt et al. 2022).

Land-use characteristics played a secondary but non-negligible role in shaping within-year abundance patterns. Forested areas, pastures, and urban land types were negatively associated with mosquito abundance, potentially reflecting reduced suitability for summer breeding or resting habitats. Nevertheless, the literature reports heterogeneous responses across landscape contexts: some studies documented lower *Cx. pipiens s.l.* abundance in highly urbanized or forested areas (Rosà et al. 2014, Roiz et al. 2015), whereas others highlighted the species’ capacity to exploit urban and peri-urban environments characterized by artificial containers, drainage systems, and favorable microclimatic conditions (Brugman et al. 2018, Amdouni et al. 2022, Giesen et al. 2023). The strong positive association observed for water bodies is consistent with previous findings from rice fields, wetlands, and irrigation networks, which are recognized as highly productive breeding habitats (Amdouni et al. 2022, Bakran-Lebl et al. 2023, Giesen et al. 2023).

From an applied perspective, these within-year results indicate that *Cx. pipiens s.l.* abundance responds rapidly to short-term temperature and rainfall anomalies, underscoring the potential value of integrating near-real-time meteorological data into adaptive surveillance and early-warning systems. Indicators such as $T_{14d}\;$and $R_{14d}$ may help anticipate within-season population peaks, informing timely adjustments in trap deployment and supporting targeted vector control and surveillance actions during periods of increased WNV transmission risk.

### Spatial Abundance

The spatial analysis identified several environmental drivers associated with long-term *Cx. pipiens s.l.* abundance. Forest cover and green urban areas showed the strongest negative associations, indicating that these landscape types are less favorable for sustaining high mosquito densities. A negative association was also observed for general urban areas, suggesting reduced suitability in highly urbanized environments.

Conversely, cumulative degree-days during the pre-activity period (DDPAP) were positively associated with spatial abundance, highlighting the importance of early-season thermal conditions in shaping long-term population levels. This finding is consistent with the role of temperature in regulating mosquito development and seasonal population build-up, supporting the role of early-season thermal accumulation in accelerating larval development and enabling earlier population establishment.

Water bodies showed a positive, although not statistically significant, association with mosquito abundance. While this trend is consistent with the known ecological role of aquatic habitats for *Cx. pipiens* s.l. (Rosà et al. 2014, Amdouni et al. 2022, Giesen et al. 2023), it should be interpreted with caution, as the confidence intervals include zero and therefore do not provide strong statistical support for this relationship. The observed pattern may reflect context-dependent effects or local environmental variability. Similarly, pastures exhibited a weak and nonsignificant association, indicating a limited or variable contribution to spatial variability in mosquito abundance.

The negative association observed for forested and urban environments is consistent with previous studies reporting reduced *Cx. pipiens* s.l. densities in less suitable or structurally constrained habitats (Rosà et al. 2014). However, the literature also highlights the species’ ecological flexibility, particularly in urban and peri-urban environments where artificial breeding sites and favorable microclimatic conditions may support mosquito populations (Brugman et al. 2018, Amdouni et al. 2022, Giesen et al. 2023). These contrasting findings suggest that the effects of land-use categories are strongly modulated by local landscape configuration and management practices.

While the present study focuses on the Po Valley, the identified drivers are broadly consistent with findings from other temperate regions, where temperature and landscape structure are recognized as key determinants of *Cx. pipiens* s.l. dynamics (Carrieri et al. 2014, Marcolin et al. 2025). However, the highly managed and irrigated landscape of the Po Valley may modulate these relationships. Therefore, although the general ecological patterns are likely transferable, their magnitude may vary depending on local environmental and land-use conditions.

From an applied perspective, these spatial patterns highlight areas potentially suitable for targeted surveillance and vector control, particularly in low-forest, low-urban, and thermally favorable environments. Integrating spatial predictions into decision-support tools may support optimized trap placement, more efficient resource allocation, and targeted vector management strategies.

### Implications for Risk Assessment, Surveillance Design, and Early Detection

The combined analysis of between-year, within-year, and spatial dynamics provides a coherent ecological framework describing how *Cx. pipiens s.l.* populations respond to climatic variability and landscape structure across Northern Italy, with direct implications for public health risk assessment, vector surveillance optimization, and early detection of WNV circulation.

The strong dependence of between-year abundance on pre-activity period temperature and precipitation anomalies indicates that the pre-season period represents a valuable predictive window for anticipating mosquito population build-up. Mild and wet pre-activity period are likely to support higher summer abundance, thereby creating favorable conditions for WNV amplification. Pre-activity periods climatic indicators (particularly cumulative precipitation) could therefore be used as early-warning metrics to guide surveillance planning prior to the transmission season.

While the identification of pre-season climatic indicators provides a useful basis for early warning, defining operational thresholds for surveillance activation would require further validation across different epidemiological and environmental contexts. These indicators should therefore be interpreted as relative signals to support adaptive surveillance strategies rather than fixed thresholds within a context-specific surveillance framework.

Within-year analyses show that *Cx. pipiens s.l.* abundance responds rapidly to short-term variability in temperature and rainfall. Integrating near-real-time meteorological information into surveillance frameworks could support adaptive monitoring strategies, enabling targeted increases in sampling effort or adult trapping during periods of elevated risk. Such information may also assist in refining the timing of early-season larviciding and guide surveillance-related actions during the initial phases of WNV circulation. However, larviciding must be implemented early to be effective, while adulticiding is often of limited applicability in rural and agricultural landscapes due to the large spatial extent of areas requiring treatment.

The spatial analysis highlighted areas where mosquito abundance is consistently higher, particularly along the Po River corridor, in irrigated agricultural landscapes and in wetland-rich municipalities. These patterns may support the spatial prioritization of surveillance activities, including increased trapping density and targeted virological monitoring. However, results should be interpreted as indicators of relative mosquito abundance rather than direct measures of transmission risk.

Finally, integrating a long-term, spatially extensive dataset (2014–2022) with complementary modeling approaches provides a robust basis for developing decision-support tools. By combining climatic forecasts, land-use information, and entomological data, such tools could generate dynamic abundance maps, seasonal predictions, and scenario-based assessments of climate-driven changes in vector dynamics, supporting timely interventions within integrated WNV surveillance programmes.

### Limitations

Despite the strengths of the long-term and spatially extensive dataset, several limitations should be acknowledged. First, the entomological surveillance design differed between regions and years in terms of spatial grid resolution, trap density, and sampling effort. However, both regional systems follow national guidelines within the Italian integrated surveillance programme, ensuring overall methodological consistency. To improve comparability, analyses were restricted to trapping sites with sufficient sampling frequency and continuity, excluding sites with interrupted monitoring. Nevertheless, some residual heterogeneity may remain and could influence comparability across space and time.

The spatial distribution of sampling sites is primarily focused on rural and peri-urban environments, reflecting the design of the national WNV surveillance programme, which targets areas where avian hosts and *Culex* mosquitoes co-occur. While this approach is consistent with the ecological characteristics of *Cx. pipiens s.l.* and surveillance objectives, highly urban environments may be under-represented.

Meteorological variables, although derived from high-resolution ERA5-Land data and refined through altimetric correction, may not fully capture microclimatic conditions at the trap level.

Overall, while these aspects do not affect the main patterns identified, they should be considered when interpreting the results and their application in operational surveillance contexts.

## Conclusions

This study provides a comprehensive assessment of the climatic and landscape drivers shaping *Cx. pipiens s.l.* population dynamics in a major West Nile virus (WNV) endemic area in Northern Italy, based on a long-term and spatially extensive dataset (2014–2022). By combining between-year, within-year, and spatial modeling approaches, the analysis shows that mosquito abundance is regulated by the interaction between pre-season hydrological conditions, short-term thermal variability, and the availability of aquatic and agricultural habitats.

At the between-year scale, pre-activity period precipitation emerged as the main predictor of mosquito abundance in the subsequent summer, emphasizing the importance of the pre-season period for population build-up. At the seasonal scale (within-year), short-term climatic fluctuations strongly influenced mosquito activity during the peak transmission period. Spatial analyses revealed clear environmental gradients, with higher abundance in irrigated agricultural landscapes, wetland-rich areas, and along the Po Valley corridor and lower levels in upland and forest-dominated regions.

Together, these findings highlight the importance of integrating multiple temporal and spatial scales when assessing WNV transmission risk. Pre-activity period climate anomalies, particularly in precipitation, may provide early indications of increased vector abundance, supporting the development of pre-season warning systems. During the transmission season, incorporating short-term weather information into surveillance workflows may improve the timing and targeting of control measures. In addition, spatially explicit information on habitat suitability can guide trap placement, optimize resource allocation, and strengthen early detection in high-risk areas.

The combined use of machine-learning models, transition analyzes and interpretability tools offers a flexible and operational framework for vector surveillance. By linking climatic information, land-use patterns, and entomological data, this approach supports more accurate risk assessment and evidence-based decision-making within integrated One Health surveillance systems for WNV.

## Supplementary Material

tjag094_Supplementary_Data
