# Supplementary material for: Meteorological and land-use determinants of Culex pipiens s.l. spatio-temporal dynamics in Northern Italy
Source: J Med Entomol. 2026 Jun 30;63(3):tjag094. doi: 10.1093/jme/tjag094 (PMC13316420; doi:10.1093/jme/tjag094)
Supplement: tjag094_Supplementary_Data [file tjag094_supplementary_data.pdf]

Appendix 1

Region	Province	Traps per year	Years per trap	Epiweek											
				23	24	25	26	27	28	29	30	31	32	33	34
Emilia-Romagna	BO	15.4 ± 0.5	7.0 ± 2.6	267.1 ± 261.8	437.7 ± 373.1	482.9 ± 1136.9	433.9 ± 350.9	307.7 ± 266.8	285.2 ± 237.1	378.9 ± 541.0	235.0 ± 183.3	144.6 ± 130.7	159.0 ± 185.6	131.3 ± 230.6	113.0 ± 143.2
	FC	3.0 ± 0.0	4.5 ± 2.6	192.6 ± 245.8	161.0 ± 101.5	206.9 ± 272.9	250.7 ± 209.1	172.5 ± 157.2	90.0 ± 50.9	139.4 ± 165.6	36.8 ± 46.3	79.8 ± 104.4	32.7 ± 29.9	75.4 ± 134.7	32.3 ± 8.1
	FE	27.2 ± 1.0	8.2 ± 2.0	247.7 ± 326.8	505.3 ± 530.0	606.0 ± 721.8	741.3 ± 1065.4	455.5 ± 513.1	519.3 ± 667.1	395.2 ± 433.0	311.6 ± 357.2	169.3 ± 196.6	124.2 ± 163.4	76.9 ± 84.8	83.5 ± 135.3
	MO	11.6 ± 0.5	8.7 ± 1.2	318.7 ± 283.2	508.4 ± 844.7	445.1 ± 386.6	619.7 ± 638.4	374.4 ± 321.5	390.2 ± 298.5	333.7 ± 313.6	312.8 ± 274.3	262.8 ± 294.7	190.8 ± 163.1	107.5 ± 146.0	133.4 ± 188.2
	PC	8.7 ± 1.7	7.8 ± 1.7	246.5 ± 340.4	520.2 ± 437.0	524.2 ± 431.2	734.4 ± 597.9	642.4 ± 489.5	510.0 ± 451.4	447.7 ± 419.9	405.1 ± 318.6	273.2 ± 325.2	243.6 ± 237.0	181.5 ± 100.5	214.5 ± 221.1
	PR	8.2 ± 1.3	6.7 ± 2.9	197.4 ± 136.6	529.5 ± 756.6	492.5 ± 547.1	577.5 ± 690.3	583.9 ± 716.9	405.0 ± 374.9	345.4 ± 326.3	252.9 ± 239.1	205.0 ± 164.0	215.6 ± 260.3	99.0 ± 92.5	104.3 ± 118.6
	RA	6.3 ± 0.5	5.7 ± 3.0	352.3 ± 419.7	247.7 ± 347.4	500.2 ± 976.0	341.6 ± 567.8	492.3 ± 633.0	165.6 ± 164.6	366.0 ± 668.9	91.3 ± 83.2	185.6 ± 143.6	49.1 ± 44.2	93.0 ± 112.6	40.3 ± 43.1
	RE	10.9 ± 1.1	7.5 ± 2.3	521.2 ± 907.4	528.3 ± 666.0	504.0 ± 577.2	613.8 ± 710.1	514.4 ± 530.4	379.9 ± 371.7	403.3 ± 395.4	265.6 ± 240.3	283.0 ± 293.0	180.6 ± 212.0	151.9 ± 126.8	92.8 ± 105.6
	RN	1.8 ± 0.4	5.3 ± 4.0	98.7 ± 118.6		82.2 ± 87.4	64.0 ± 83.4	75.3 ± 56.9	32.0 ± 41.0	74.6 ± 53.8	84.0 ± 116.0	93.6 ± 85.7	54.0 ± 73.5	67.5 ± 79.7	12.5 ± 13.4
Lombardy	BG	3.6 ± 0.9	5.3 ± 3.5	113.0 ± 0.0	41.2 ± 46.7	67.6 ± 110.6	64.2 ± 88.1	77.0 ± 87.5	76.3 ± 76.8	94.4 ± 147.2	25.5 ± 31.9	90.4 ± 136.9	89.1 ± 107.6	68.4 ± 124.1	36.2 ± 49.5
	BS	6.6 ± 0.7	4.2 ± 2.5	155.9 ± 178.0	286.3 ± 441.6	225.1 ± 228.3	203.8 ± 176.7	267.7 ± 446.7	216.9 ± 205.3	131.0 ± 140.8	144.9 ± 139.0	68.5 ± 93.7	151.9 ± 157.9	145.9 ± 123.0	136.8 ± 132.0
	CO	1.6 ± 0.5	2.0 ± 1.4	18.7 ± 32.3	4.0 ± 4.6	31.3 ± 15.2	35.0 ± 29.7		51.5 ± 70.7	63.0 ± 91.9	7.3 ± 7.8	38.0 ± 48.8	13.2 ± 17.6	40.0 ± 0.0	23.0 ± 32.5
	CR	2.9 ± 0.9	2.5 ± 2.1	273.0 ± 243.9	350.0 ± 533.7	325.4 ± 258.1	275.2 ± 263.9	47.0 ± 0.0	179.1 ± 175.3	215.5 ± 160.9	175.9 ± 164.0		125.3 ± 125.9		153.9 ± 195.4
	LC	1.2 ± 0.5	1.7 ± 1.2	287.0 ± 403.1		129.5 ± 113.8	166.0 ± 0.0	140.5 ± 106.0		142.5 ± 127.4	6.0 ± 0.0	47.3 ± 36.5		0.0 ± 0.0	77.3 ± 36.6
	LO	3.0 ± 0.0	5.4 ± 2.1	127.8 ± 105.9	240.2 ± 286.1	158.9 ± 103.3	288.1 ± 271.5	172.9 ± 90.7	329.3 ± 368.6	227.1 ± 191.3	165.2 ± 117.4	101.8 ± 103.7	216.8 ± 350.5	88.2 ± 56.6	71.8 ± 66.1
	MB	1.0 ± 0.0	5.0 ± 0.0	3.0 ± 2.0		6.5 ± 6.4	4.3 ± 1.5	2.0 ± 1.4	2.0 ± 1.4	4.0 ± 7.3	0.0 ± 0.0	2.5 ± 2.1	4.0 ± 4.2	1.0 ± 1.4	6.5 ± 0.7
	MI	5.2 ± 0.4	3.4 ± 2.1	69.8 ± 81.9	43.2 ± 61.4	106.3 ± 107.6	79.2 ± 133.7	172.5 ± 177.1	245.7 ± 389.5	130.5 ± 136.1	54.0 ± 69.4	90.0 ± 91.7	108.7 ± 208.4	71.8 ± 79.4	55.1 ± 60.2
	MN	7.4 ± 0.7	4.5 ± 2.8	238.2 ± 447.9	189.3 ± 348.7	432.9 ± 497.5	333.2 ± 410.3	391.8 ± 396.9	202.0 ± 250.9	360.8 ± 471.6	228.9 ± 264.8	200.1 ± 273.7	185.5 ± 317.3	83.7 ± 135.6	32.9 ± 30.6
	PV	6.3 ± 1.1	5.2 ± 3.3	60.0 ± 99.9	287.0 ± 0.0	147.2 ± 225.1	447.2 ± 540.5	307.7 ± 369.2	516.0 ± 413.3	409.7 ± 474.8		208.4 ± 321.4	257.3 ± 318.7	121.4 ± 125.2	136.8 ± 248.8
	SO	2.5 ± 1.0	2.5 ± 1.0	0.7 ± 1.2	2.0 ± 1.0	15.4 ± 35.1	3.7 ± 3.5	12.6 ± 11.1	7.3 ± 6.4	8.5 ± 10.1	1.7 ± 1.5	13.0 ± 16.3		10.3 ± 2.1	1.0 ± 1.4
	VA	1.0 ± 0.0	4.0 ± 0.0	12.7 ± 12.2	9.0 ± 2.8	3.0 ± 0.0	79.0 ± 82.0		11.0 ± 9.9	33.0 ± 18.4	124.5 ± 123.7	34.0 ± 30.5	144.0 ± 0.0	47.0 ± 0.0	13.3 ± 9.3

Table A1. Summary of *Culex pipiens sl* surveillance effort and intra-seasonal abundance across the studied provinces in Emilia-Romagna and Lombardy. The table reports the mean number of active traps per year, the mean number of monitored years per trap, and the weekly mean abundance (number of mosquitoes per trap) from epidemiological weeks 23 to 34. Values are expressed as mean ± standard deviation (SD).

		Prediction		
		**A1**	**A2**	**A3**
**Observed**	**A1**	218	83	20
	**A2**	80	152	88
	**A3**	23	85	212

Table A2. Confusion matrix of the Gradient Boosting model for between-year dynamics of *Cx. pipiens sl* abundance. Rows represent observed abundance classes, while columns represent predicted classes. Abundance classes (A1–A3) correspond to tertiles of summer mean abundance (A): low (A1), medium (A2), and high (A3).

		Prediction		
		**A1**	**A2**	**A3**
**Observed**	**A1**	225	63	33
	**A2**	104	86	130
	**A3**	27	50	243

Table A3. Confusion matrix of the Gradient Boosting model for within-year dynamics (June–July transition) of *Cx. pipiens sl* abundance. Rows represent observed abundance classes, while columns represent predicted classes. Abundance classes (A1–A3) correspond to tertiles of monthly mean abundance (A): low (A1), medium (A2), and high (A3).

		Prediction		
		**A1**	**A2**	**A3**
**Observed**	**A1**	159	68	9
	**A2**	66	108	61
	**A3**	23	59	136

Table A4. Confusion matrix of the Gradient Boosting model for within-year dynamics (July–August transition) of *Cx. pipiens sl* abundance. Rows represent observed abundance classes, while columns represent predicted classes. Abundance classes (A1–A3) correspond to tertiles of monthly mean abundance (A): low (A1), medium (A2), and high (A3).
